# Recurrent ischemic stroke with patent foramen ovale linked to seronegative antiphospholipid syndrome: a case report and literature review

**DOI:** 10.3389/fimmu.2025.1558309

**Published:** 2025-04-02

**Authors:** Xin Zhou, Xiaoxiong Huang, Leliang Zhou, Yongbiao Zou

**Affiliations:** Department of Neurology, The Central Hospital of Shaoyang, Shaoyang, Hunan, China

**Keywords:** seronegative antiphospholipid syndrome (SNAPS), non-criteria antiphospholipid antibodies, recurrent ischemic stroke, patent foramen ovale, antithrombotic therapy

## Abstract

The disability and mortality rates of recurrent ischemic stroke tend to be higher than those of the first stroke, which seriously affects the quality of life and prognosis of patients. Accurately identifying the etiology of stroke is critical for guiding effective treatment, and one of the most noteworthy in young patients is antiphospholipid syndrome (APS). Despite the progress made in APS research, seronegative APS (SN-APS) remains underdiagnosed. We present a case of recurrent ischemic stroke accompanied by PFO, which is associated with SN-APS. A 49-year-old female presented with expressive dysphasia and unresponsiveness on October 4, 2022. The magnetic resonance imaging (MRI) of cerebral revealed left frontal lobe infarcts; neither magnetic resonance angiography (MRA) nor magnetic resonance venography (MRV) showed significant hemodynamic stenosis or venous thrombosis. Subsequently, she was admitted to global aphasia and right hemiparesis and treated with intravenous thrombolysis and PFO closure on July 21, 2023. 9 months later, on April 23, 2024, she had a recurrence of ischemic stroke and tested negative for conventional antiphospholipid antibodies (aPL). However, the livedo reticularis on the inner side of the patient’s feet triggered our in-depth investigations and reflections. Due to the presence of positive non-criteria antibodies, the patient was eventually diagnosed with SN-APS and received standardized antithrombotic therapy. Our report suggests stroke can be a major manifestation of SN-APS, and a comprehensive evaluation by rheumatology and neurology teams is crucial to recognize it early. Prompt diagnosis and early anticoagulation therapy are beneficial to the prognosis of patients.

## Introduction

Antiphospholipid syndrome (APS) is well known as an autoimmune thrombophilia disease defined by the presence of venous and/or arterial thrombotic events, pregnancy morbidity, and mild thrombocytopenia (1). Patients have persistent positive tests for antiphospholipid antibodies(aPL), such as anticardiolipin antibodies (aCL), anti-beta 2 glycoprotein-I (aβ2GPI), and lupus anticoagulant (LAC) (1). APS is classified as primary (PAPS) when occurring in isolation and secondary (SAPS) when associated with other autoimmune disorders, primarily systemic lupus erythematosus (SLE) (1). To date, a growing number of studies have demonstrated that patients may present clinical features strongly suggestive of APS but with negative titers of aPL (2). They meet the diagnosis of seronegative APS (SN-APS) and are further treated with antithrombotic therapy (2, 3).

As a prothrombotic autoimmune disorder, APS has a severe impact on several vital organs, including the central nervous system (CNS), cardiac, lung and renal. Acute ischemic stroke (AIS) and transient ischemic attack (TIA) are thought to be the most common events related to arterial pathology in APS, particularly in younger patients (1). It has been reported that 20% of strokes in young individuals are associated with APS (4). AIS induced by the presence of thromboembolic episodes is not only catastrophic for the patient but also burdensome for the family. Therefore, recognition of APS in patients with neurological symptoms as early as possible is critical to facilitate appropriate treatment and improve outcomes. However, the clinical significance of non-criteria antibodies in stroke etiology remains unclear. Here we encountered a case of recurrent ischemic stroke with patent foramen ovale (PFO) that displayed typical features of APS but tested negative for classical aPL.

## Case presentation

**Figure 1:**
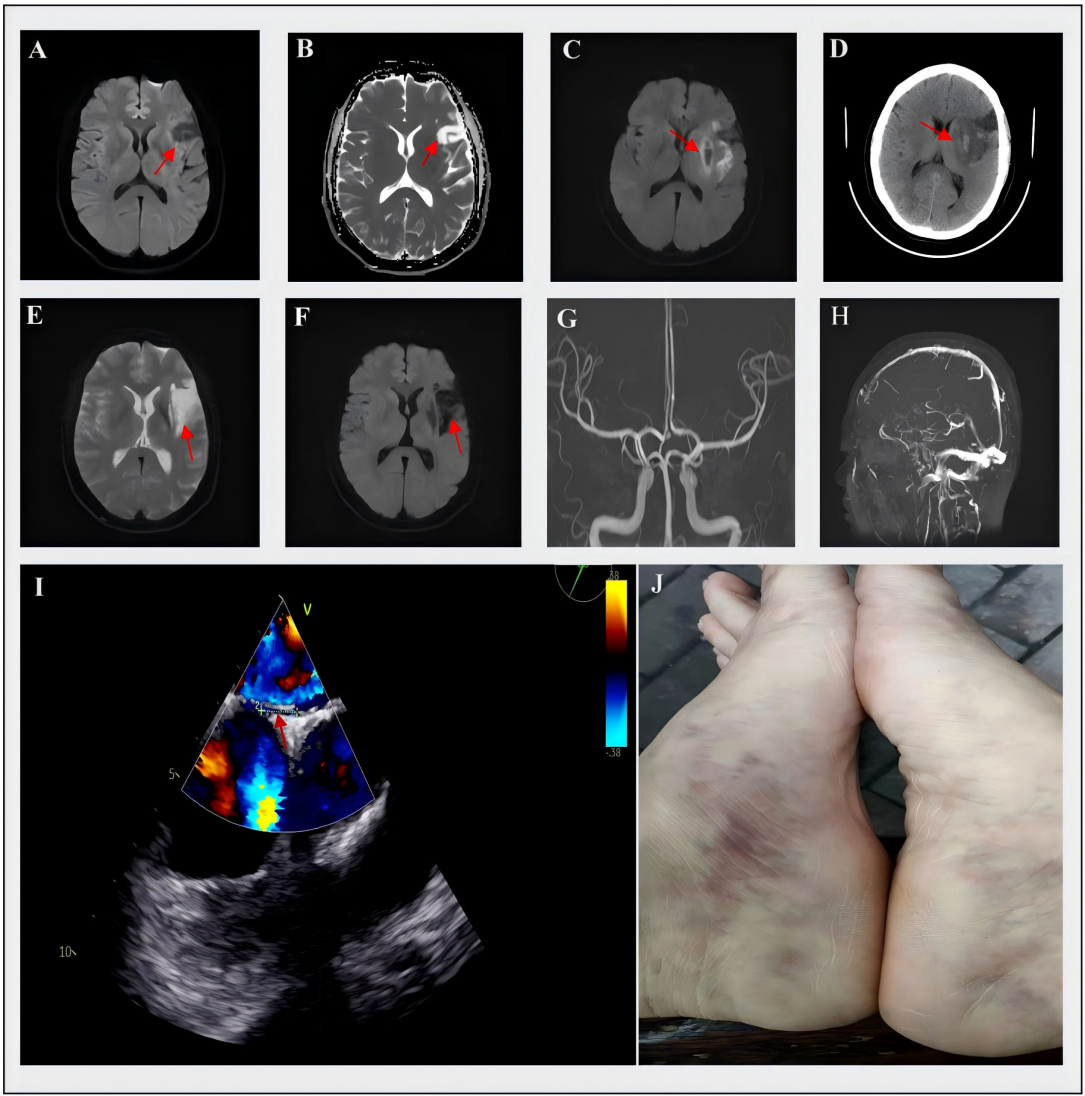
Cerebral MRI, MRA, MRV and TEE of the patient. **(A, B)** On October 4, 2022, the MRI showed hypointensity in the left frontal lobe on DWI and hyperintensity in ADC images; **(C)** On July 21, 2023, the MRI demonstrated hyperintensity in the left temporal insular lobe and patchy hypointensity in the basal ganglia on DWI, **(D)** On the same day, the CT showed hyperdensity in the basal ganglia with an increasing area of hypodensity in surrounding regions; **(E)** On April 23, 2024, the MRI detected a new lesion with hyperintensity in the left corona radiata on DWI, **(F)** The ADC images showed hypointensity of this new lesion; **(G, H)** showed the normal results on the MRA and MRV; **(I)** the result of TEE and the arrow indicated the presence of PFO; **(J)** The livedo reticularis on the inner side of the patient’s feet was clearly visible. DWI, diffusion-weighted imaging; ADC, apparent diffusion coefficient; MRI, magnetic resonance imaging; MRA, magnetic resonance angiography; MRV, magnetic resonance venography; TEE, transesophageal echocardiogram; PFO, patent foramen ovale.

A 49-year-old woman with a history of migraine presented to our outpatient clinic on October 4, 2022, with 20 days of difficulty finding words and unresponsiveness. She had no classical cerebrovascular risk factors, such as hypertension, dyslipidemia, diabetes and smoking, and denied the history of thrombosis or recurrent pregnancy loss. The examination revealed that her expression was apathetic, both memory and orientation were decreased (Minimum Mental State Examination (MMSE) scale 23 scores); she had neither limb weakness nor facial asymmetry. Her vital signs were normal, as follows: body temperature, 36.7°C; heart rate, 80 beats/min; respiration, 18 beats/min, and blood pressure, 122/76 mmHg. The cerebral magnetic resonance imaging (MRI) showed a chronic cortical infarct in the left frontal lobe (**Figures 1A, B**). The magnetic resonance angiography (MRA) (**Figure 1G**) and ultrasound sonography of carotid arteries showed no stenosis or occlusion of the intracranial or extracranial arteries. Due to her refusal of hospitalization for further evaluation, the patient was treated with daily aspirin and atorvastatin.

The patient was hospitalized on July 21, 2023, with sudden-onset right limb weakness and dysarthria for 4 hours. The admission examination showed somnolence, global aphasia, right facial palsy, right hypoesthesia and hemiparesis (Medical Research Council (MRC) grade 3/5), and positive pathological signs on the right side, with normal vital signs. The National Institutes of Health Stroke Scale (NIHSS) score was 11. Due to the consideration of ischemic stroke and the absence of contraindications, she was treated by intravenous thrombolytic therapy with 50 mg alteplase. After alteplase infusion, the patient’s neurological symptoms were partially improved (NIHSS score of 7), but the subsequent MRI and CT scan exhibited that there were left temporal insular lobe infarcts with small hemorrhagic transformation of the basal ganglia (about 2ml) (**Figures 1C, D**). There were no significant venous thrombosis or arteriovenous malformations on magnetic resonance venography (MRV) (**Figure 1H**). Her laboratory studies showed within normal parameters. Electrocardiogram (ECG), lower-extremity bilateral Doppler ultrasound, and chest and abdominal CT showed no abnormalities; however, further transesophageal echocardiogram (TEE) demonstrated the existence of PFO (approximately 3 mm size) (shown in **Figure 1I**), transthoracic echocardiography (TTE) assessed left ventricular ejection fraction exceeding 70%, and normal sizing in all four chambers without atrial septal aneurysms. We considered that the presence of paradoxical embolism triggered cryptogenic stroke and performed transcatheter PFO closure surgery after hemorrhage absorption. At discharge, the patient achieved good functional outcomes, was able to walk independently (mRS 1), and maintained dual antiplatelet and atorvastatin.

Surprisingly, 9 months after the second stroke, the patient was re-admitted for recurrent right hemiplegia and slurred speech on April 23, 2024. Neurological examination revealed alert mental status and the right hemiplegia (MRC grade, 2/5), indicating a NIHSS score of 9. Cerebral MRI revealed acute infarct in the left corona radiata (**Figures 1E, F**). Given that the time window for thrombolysis had expired and considering the previous intracranial hemorrhage, intravenous thrombolysis was not performed this time. Just like last time, her routine laboratory results were unremarkable, and follow-up TEE did not show any shunt. The discovery of livedo reticularis on the patient’s feet during the third admission prompted re-evaluation of the underlying etiology for her recurrent cerebral embolism (shown in **Figure 1J**). To further explore the primary embolic sources, a series of thromboembolic laboratory tests were conducted, including aCL, aβ2GPI, LAC, protein C (PC), protein S (PS), antithrombin III activity (AT-III), antinuclear antibody and antineutrophil cytoplasmic antibodies (**Table 1**). Although only aβ2GPI IgA was positive, it raised our suspicions of APS. Then, we performed an immunological examination for non-criteria aPL, and the antibodies to vimentin/CL complex and anti-annexin A5 antibody were positive, which indicated the diagnosis of SN-APS (**Table 1**). Considering the high risk of recurrence of thrombotic events in the patient, long-term use of low-dose aspirin and warfarin was recommended, with an international normalized ratio (INR) range between 2-3. We followed the patient since she received intense anticoagulation and antiplatelet therapies. As for the time for submission of the manuscript, the patient’s stroke symptoms have not recurred.

**Table 1:** Result of laboratory antibody testing.

Antibodies index	Result	Reference Range
anticardiolipin antibodies (aCL)	(-)	(-)
lupus anticoagulant (LAC)	(-)	(-)
antibodies to vimentin/CL complex	16.36	0.00-10.00 (ng/ml)
anti-β2GPI antibodies (aβ2GPI) lgA	53.45	0.00-20.00 (U/mL)
anti-β2GPI antibodies (aβ2GPI) lgM	1.35	0.00-20.00 (U/mL)
anti-β2GPI antibodies (aβ2GPI) lgG anti-phosphatidylserine-prothrombin (aPS/PT) antibodies IgG anti-phosphatidylserine-prothrombin (aPS/PT) antibodies IgM	<0.50 29.70 16.41	0.00-20.00 (U/mL) 0.00-30.00(U/mL) 0.00-30.00(U/mL)
antibodies to vimentin/CL complex	16.36	0.00-10.00 (ng/ml)
anti-annexin A5 antibody	49.17	0.00-40.00 (ng/ml)
anti-annexin A2 antibody	18.84	0.00-40.00 (ng/ml)
anti-phosphatidylethanolamine antibody	12.12	0.00-15.00 (ng/ml)
protein C (PC)	108	70.0-120.0 (%)
protein S (PS)	65	63.5-149.0 (%)
antithrombin III activity (AT-III)	115.80	80.00-130.00 (%)
antineutrophil cytoplasmic antibodies	(-)	(-)
antinuclear antibody	(-)	(-)

**Figure 2:**
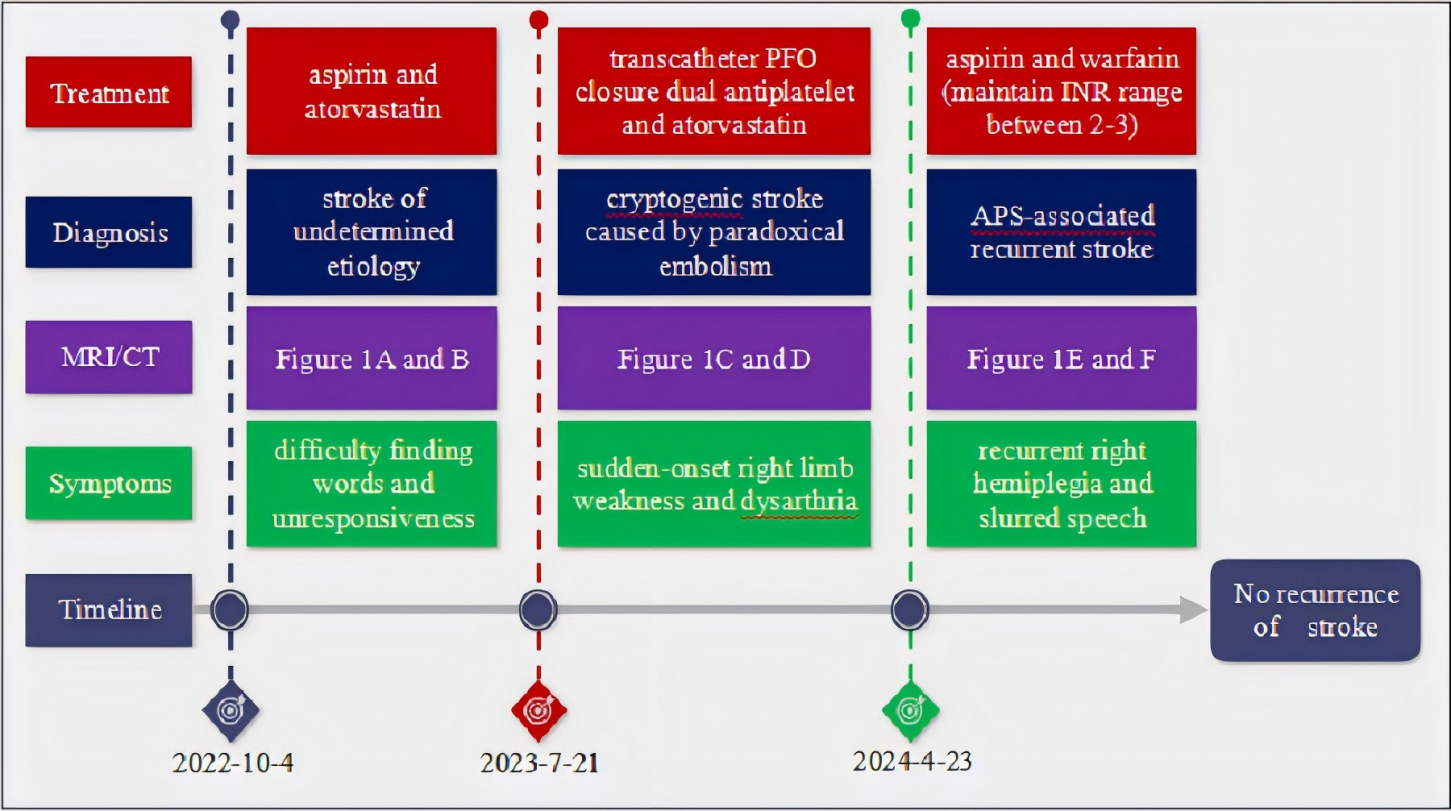
The disease duration in this case.

The timeline of this case is summarized in **Figure 2**. This clinical case highlights the diagnostic challenges posed by SN-APS, where classical aPL testing yields negative results despite thrombotic manifestations.

## Discussion

We described a rare case of frequent cerebral infarctions in the middle cerebral artery (MCA) territory, which are unusual in large-artery atherosclerosis and small-vessel occlusion, two of the most common stroke etiologies. Thromboembolic stroke was initially thought to be caused by paradoxical embolism through the PFO. However, after PFO closure, there was still a strong propensity to embolism, which inspired us to look for the underlying cause. The patient was ultimately diagnosed with primary SN-APS based on her livedo reticularis and positive non-criteria antibodies, and both the recurrent ischemic stroke and PFO were likely associated with APS. Notably, the patient’s PFO likely interacted with her hypercoagulable state to promote paradoxical embolism. This highlights the need for comprehensive evaluation of both mechanical and thrombophilic risk factors in stroke etiology. Importantly, this case underscores the need for rheumatology-neurology collaboration in stroke evaluation.

Classical APS is characterized by the presence of persistently high levels of aPL that predispose the individual to thrombotic events (5). The most common arterial thrombotic events involve AIS or TIA, and deep vein thrombosis (DVT) and pulmonary embolism (PE) are the most common venous events in patients with APS (5). Other manifestations include recurrent miscarriage, thrombocytopenia, livedo reticularis, valvulopathy, nephropathy, and so on (5). However, an increasing number of cases have reported patients with clinical manifestations highly suggestive of APS, despite their negative aPL test results (5, 6). For these patients, the diagnosis of SN-APS has been proposed in recent years, and non-criteria antibodies are recognized as pathogenic or involved in the occurrence of thrombotic events (2, 6). Nowadays, growing evidence has emphasized the potential role of non-criteria antibodies in the diagnosis of SN-APS, including anti-phosphatidylserine-prothrombin (aPS/PT) antibodies, antibodies against the domain I of β2-glycoprotein (aDI), IgA aCL and aβ2GPI, antibodies to vimentin/CL complex, anti-annexin V antibodies/annexin A5 resistance (6). For instance, recent well-documented research shows a significant correlation between aPS/PT antibodies and both thrombosis and pregnancy-related morbidities (7, 8). What’s more, two prospective studies have confirmed the role of aPT in predicting the risk of first-time or recurrent thrombosis in APS patients (9, 10). Meanwhile, proteomic studies identify the vimentin/CL complex as a potential APS antigen target, also detected in SN-APS patients (11). Apart from that, a 5-year case-control study of 244 asymptomatic patients screened for aPL showed that the presence of IgA a-β2GPI is associated with an increased risk of thrombotic events in APS (12). Annexin A5 resistance is regarded as one of the antiphospholipid syndrome’s pathogenesis mechanisms. Some studies indicate that reduced annexin (13). In addition, the presence of multiple annexin autoantibodies in a patient with recurrent miscarriage, stroke, and SN-APS was documented in a case report (14). Although the clinical relevance of non-criteria antibodies in SN-APS has not been fully elucidated, related research is still evolving.

Among young adults under the age of 50, 17% of stroke and 12% of TIA are related to aPL, suggesting APS plays a significant role in the etiology of stroke in the young (15). Neuroimaging typically reveals cortical infarction, cortical atrophy, and microhemorrhages, and lesions most commonly involve the middle cerebral artery (MCA) territory. Due to vascular injury and the direct effects of antibodies on neurons and ependymal cells, neurological manifestations also include a variety of non-standard symptoms, such as migraine, epilepsy, bipolar disorder, transverse myelitis, dementia, chorea, cognitive impairment, and multiple sclerosis (16). It is worth mentioning that migraine is the most common neurological manifestation and has been observed in a great number of patients with thrombotic events, TIA or stroke (16). However, at present, no literature has clearly elaborated on the specific pathological mechanisms between APS-related thromboembolism and migraine.

Among the various causes of stroke, APS is one of the most representative underlying etiologies and is easily misdiagnosed in diagnosis because of its rarity. The patients with APS, who produce paradoxical emboli through the PFO and develop into recurrent stroke, are even less common. After searching the PubMed and Web of Science databases, we found 7 patients with APS-related ischemic stroke who had PFO, and all these patients were treated with anticoagulation (**Table 2**) (17–23). Recent studies have suggested that PFO is more commonly found in patients with thrombotic disorders such as APS, while aPL is more prevalent among patients with PFO embolism (24). PFO significantly increases the risk of recurrence and in an acquired hypercoagulable state, such as APS, there is a greater risk of paradoxical embolism (24). Although the relationship between APS and PFO has not been fully studied, PFO closure and postoperative antithrombotic therapy are indispensable for secondary prevention. The presence of PFO indicates a high risk of paradoxical embolism, which emphasizes the importance of detecting PFO in patients with APS for treatment and prognosis. However, there are different opinions on PFO closure and the majority (41.9%) advocate PFO closure with long-term anticoagulation. Several observational studies revealed that thrombophilia patients treated by PFO closure and postoperative antithrombotic therapy remained free from recurrence, while recurrent events occurred among patients who only used warfarin (24). For patients with thrombotic diseases and a prior PFO-associated stroke, the Society for Cardiovascular Angiography and Interventions (SCAI) guideline panel recommends PFO closure in addition to lifelong anticoagulation therapy rather than anticoagulation therapy alone (25). Considering issues such as stroke recurrence, bleeding, medication interruption, and non-compliance, the panel believes that thrombotic patients may experience greater risk reduction after PFO closure.

**Table 2:** Summary of clinical characteristics in reported case series of APS-associated ischemic stroke and PFO.

No.	Country	Publication date	Age (years) /Sex	Onset symptoms	MRI/CT findings	Antibodies	Treatment
1	Japan (17)	2000	68/male	left hemiplegia and consciousness	in right MCA territory region	ANA and LAC	intravenous thrombolytic, warfarin
2	Japan (18)	2004	42/female	pain of bilateral lower extremities and left-sided homonymous hemianopsia	right occipital lobe	LAC and aβ2GPI	low molecular weight heparin and warfarin
3	Britain (19)	2008	43/male	NA	left parietal lobe infarct	LAC and aCL	PFO closure, heparin and warfarin
4	Eastern European (20)	2015	34/male	left homonymous hemianopia, left sided neglect and hemiplegia	infarct in right MCA territory	aCL and aβ2GPI	intravenous thrombolytic, thrombectomy, PFO closure and warfarin
5	Mexico (21)	2019	56/female	right hand and face numbness	postcentral gyrus of the left parietal lobe	aCL	apixaban
6	Brazil (22)	2022	31/female	hemiplegia	in MCA territory region	aCL	intravenous thrombolytic, thrombectomy, PFO closure and aspirin+ warfarin
7	American (23)	2024	31/female	irritability, right-sided facial drooping and weakness	left MCA territory infarct	aβ2GPI IgG and aCL IgG	thrombectomy, aspirin+ apixaban

MCA, middle cerebral artery; ANA, anti-nuclear antibody; aCL, anticardiolipin antibodies; LAC, lupus anticoagulant; aβ2GPI, anti-beta 2 glycoprotein-I; NA, not available.

Although understanding of the pathogenesis of APS has increased in recent years, the principles and strategies for the clinical management of APS have not undergone revolutionary changes. In patients with SN-APS, treatment is particularly challenging as non-criteria antibodies are associated with various thrombotic complications, necessitating individualized risk stratification. However, research indicates that, compared with atrial fibrillation (AF) patients, there are more considerations regarding anticoagulant therapy in APS patients (26). In cases where warfarin therapy is ineffective or recurrent thrombosis occurs, two possible treatment approaches can be considered. The first is to adopt a more intensive warfarin therapy with a target international normalized ratio (INR) of 3-4 (1, 27). However, given that it is not strongly associated with a reduced risk of thrombosis in most patients, this is not the current standard practice. The second approach is to add low-dose aspirin (LDA) to the anticoagulant therapy (1, 2, 27). Nevertheless, this should be used only in high-risk patients, especially after an arterial thromboembolic event. According to the updated European League Against Rheumatism (EULAR) recommendations for APS management, for high-risk patients, particularly those who have experienced an arterial thrombotic event, the addition of low-dose aspirin (LDA) to warfarin therapy with a target INR of 2-3 was recommended (27). Vitamin K antagonists remain the most suitable treatment for most patients with thrombotic antiphospholipid syndrome. Although non-vitamin K antagonist oral anticoagulants (NOACs) have been studied in patients with APS, the results have been inconsistent, and strong evidence is still lacking (28).

Previous research has demonstrated that higher levels of aPL are associated with significant cerebral inflammation and thrombosis, yet the underlying mechanisms responsible for thrombosis in APS remain unclear (29). Some evidence has indicated that aPL could activate vascular endothelial cells, which facilitate platelet adhesion, thereby leading to a hypercoagulable state that contributes to thrombosis in patients with APS (29). Moreover, β2-GPI and oxidized low-density lipoprotein (oxLDL) combine to form complexes that are involved in vascular injury and oxidative stress and play a significant role in autoimmune-mediated atherosclerosis (29). Building on these mechanistic insights, the detection of non-criteria antibodies may help guide antithrombotic treatment strategies for arterial or venous thrombosis in SN-APS patients. Further research is critical to standardize management strategies for SN-APS and thrombotic complications. The role of non-criteria antibodies in “seronegative” patients requires confirmation through large-scale prospective clinical studies. Future areas of interest include the establishment of SN-APS-specific criteria, ongoing improvements in optimal laboratory practices for anticoagulated patients, and more nuanced recommendations for identifying patients most likely to benefit from alternatives to warfarin.

Our report has some limitations. First, since SN-APS is usually a diagnosis of exclusion, we did not conduct at least two consecutive tests to confirm the negativity of classical aPL. Second, after the patient was diagnosed with SN-APS, we neglected to re-examine the non-criteria antibodies titers at an interval of at least 12 weeks, although not all patients remain positive over time.

## Conclusion

In summary, APS remains a complex and multifaceted condition that severely impacts morbidity and quality of life. This case advocates the addition of non-criteria antibody testing in recurrent stroke, particularly in the presence of vascular cutaneous manifestations like livedo reticularis. The clinical diagnosis of APS is often difficult and easily missed, not only because it is a rare disease but also due to gaps in knowledge of its antibodies and manifestations. Our report suggests SN-APS should be considered in patients exhibiting recurrent stroke, even though classical antibody results are negative. Additionally, we highlight that it is crucial to recognize APS early and adopt the most appropriate antithrombotic therapy to reduce the rate of thrombosis recurrence in patients with APS.

## Data Availability

The original contributions presented in the study are included in the article/**Supplementary Material**. Further inquiries can be directed to the corresponding author.

## References

[1] Garcia D Erkan D. Diagnosis and management of the antiphospholipid syndrome. N Engl J Med. (2018) 378:2010–21. doi: 10.1056/NEJMra1705454 29791828

[2] Pignatelli P Ettorre E Menichelli D Pani A Violi F Pastori D. Seronegative antiphospholipid syndrome: refining the value of “non-criteria” antibodies for diagnosis and clinical management. Haematologica. (2020) 105:562–72. doi: 10.3324/haematol.2019.221945 PMC7049333 32001534

[3] Hughes GR Khamashta MA. Seronegative antiphospholipid syndrome. Ann Rheum Dis. (2003) 62:1127. doi: 10.1136/ard.2003.006163 14644846 PMC1754381

[4] Cervera R Serrano R Pons-Estel GJ Ceberio-Hualde L Shoenfeld Y de Ramón E. Morbidity and mortality in the antiphospholipid syndrome during a 10-year period: a multicentre prospective study of 1000 patients. Ann Rheum Dis. (2015) 74:1011–8. doi: 10.1136/annrheumdis-2013-204838 24464962

[5] Schreiber K Sciascia S de Groot PG Devreese K Jacobsen S Ruiz-Irastorza G. Antiphospholipid syndrome. Nat Rev Dis Primers. (2018) 4:18005. doi: 10.1038/nrdp.2018.5 29368699

[6] Nayfe R Uthman I Aoun J Saad Aldin E Merashli M Khamashta MA. Seronegative antiphospholipid syndrome. Rheumatol (Oxford). (2013) 52:1358–67. doi: 10.1093/rheumatology/ket126 23502076

[7] Shi H Zheng H Yin YF Hu QY Teng JL Sun Y. Antiphosphatidylserine/prothrombin antibodies (aPS/PT) as potential diagnostic markers and risk predictors of venous thrombosis and obstetric complications in antiphospholipid syndrome. Clin Chem Lab Med. (2018) 56:614–24. doi: 10.1515/cclm-2017-0502 29166262

[8] Žigon P Podovšovnik A Ambrožič A Tomšič M Hočevar A Gašperšič N. Added value of non-criteria antiphospholipid antibodies for antiphospholipid syndrome: lessons learned from year-long routine measurements. Clin Rheumatol. (2019) 38:371–8. doi: 10.1007/s10067-018-4251-7 30099654

[9] Forastiero R Martinuzzo M Pombo G Puente D Rossi A Celebrin L. A prospective study of antibodies to beta2-glycoprotein I and prothrombin, and risk of thrombosis. J Thromb Haemost. (2005) 3:1231–8. doi: 10.1111/j.1538-7836.2005.01295.x 15946213

[10] Bizzaro N Ghirardello A Zampieri S Iaccarino L Tozzoli R Ruffatti A. Anti-prothrombin antibodies predict thrombosis in patients with systemic lupus erythematosus: a 15-year longitudinal study. J Thromb Haemost. (2007) 5:1158–64. doi: 10.1111/j.1538-7836.2007.02532.x 17388963

[11] Ortona E Capozzi A Colasanti T Conti F Alessandri C Longo A. Vimentin/cardiolipin complex as a new antigenic target of the antiphospholipid syndrome. Blood. (2010) 116:2960–7. doi: 10.1182/blood-2010-04-279208 20634382

[12] Tortosa C Cabrera-Marante O Serrano M Martínez-Flores JA Pérez D Lora D. Incidence of thromboembolic events in asymptomatic carriers of IgA anti ß2 glycoprotein-I antibodies. PLoS One. (2017) 12:e0178889. doi: 10.1371/journal.pone.0178889 28727732 PMC5519006

[13] Wolgast LR Arslan AA Wu XX Beyda JN Pengo V Rand JH. Reduction of annexin A5 anticoagulant ratio identifies antiphospholipid antibody-positive patients with adverse clinical outcomes. J Thromb Haemost. (2017) 15:1412–21. doi: 10.1111/jth.13699 28393472

[14] Scholz P Auler M Brachvogel B Benzing T Mallman P Streichert T. Detection of multiple annexin autoantibodies in a patient with recurrent miscarriages, fulminant stroke and seronegative antiphospholipid syndrome. Biochem Med (Zagreb). (2016) 26:272–8. doi: 10.11613/issn.1846-7482 PMC4910275 27346975

[15] Sciascia S Sanna G Khamashta MA Cuadrado MJ Erkan D Andreoli L. The estimated frequency of antiphospholipid antibodies in young adults with cerebrovascular events: a systematic review. Ann Rheum Dis. (2015) 74:2028–33. doi: 10.1136/annrheumdis-2014-205663 24942381

[16] Miesbach W Gilzinger A Gökpinar B Claus D Scharrer I. Prevalence of antiphospholipid antibodies in patients with neurological symptoms. Clin Neurol Neurosurg. (2006) 108:135–42. doi: 10.1016/j.clineuro.2005.03.005 16412834

[17] Okura H Tomon M Nishiyama S Yoshikawa T. Patent foramen ovale and “catastrophic” antiphospholipid syndrome. Intern Med. (2000) 39:83. doi: 10.2169/internalmedicine.39.83 10674859

[18] Ago T Ooboshi H Kitazono T Imamura T Takada J Ibayashi S. Brain infarction associated with antiphospholipid antibody syndrome caused by paradoxical embolism through patent foramen ovale. J Neurol. (2004) 251:757–9. doi: 10.1007/s00415-004-0431-2 15311357

[19] Williamson JML Dalton RSJ Chester JF. Popliteal venous aneurysm causing pulmonary embolism and paradoxical embolisation in a patient with antiphospholipid syndrome. Eur J Vasc Endovasc Surg. (2008) 36:227–9. doi: 10.1016/j.ejvs.2008.03.005 18485755

[20] Osuafor C Murphy S. Stroke recurrence after percutaneous patent foramen ovale closure: A case of undiagnosed antiphospholipid syndrome. Int J Stroke. (2015) 10:23–4. doi: 10.1111/ijs.12634_6

[21] Gerstein NS Clegg SD Levin DB Fish AC Tolstrup K Nakanishi K. A case-based discussion on the management of cryptogenic stroke and patent foramen ovale in the patient with a hypercoagulable disorder. J Cardiothorac Vasc Anesth. (2019) 33:3476–85. doi: 10.1053/j.jvca.2019.07.136 31473116

[22] MaChado P. Antiphospholipid syndrome causing two rare events in a woman at different times: libman-sacks mitral valvulopathy and paradoxical emboli with a stroke. J Am Coll Cardiol. (2022) 79:3461. doi: 10.1016/S0735-1097(22)04452-7

[23] Jamal F Kumar R Hakobyan N. Elucidating the neuropsychiatric phenomena of antiphospholipid syndrome in a 31-year-old female. Cureus. (2024) 16:e64856. doi: 10.7759/cureus.64856 39156266 PMC11330320

[24] Tanaka Y Ueno Y Miyamoto N Shimada Y Tanaka R Hattori N. Patent foramen ovale and atrial septal aneurysm can cause ischemic stroke in patients with antiphospholipid syndrome. J Neurol. (2013) 260:189–96. doi: 10.1007/s00415-012-6613-4 22836909

[25] Kavinsky CJ Szerlip M Goldsweig AM Amin Z Boudoulas KD Carroll JD. SCAI guidelines for the management of patent foramen ovale. J Soc Cardiovasc Angiogr Interv. (2022) 1:100039. doi: 10.1016/j.jscai.2022.100039 39131947 PMC11307505

[26] Pastori D Parrotto S Vicario T Saliola M Mezzaroma I Violi F. Antiphospholipid syndrome and anticoagulation quality: a clinical challenge. Atherosclerosis. (2016) 244:48–50. doi: 10.1016/j.atherosclerosis.2015.10.105 26584138

[27] Barbhaiya M Zuily S Naden R Hendry A Manneville F Amigo MC. The 2023 ACR/EULAR antiphospholipid syndrome classification criteria. Arthritis Rheumatol. (2023) 75:1687–702. doi: 10.1002/art.v75.10 37635643

[28] Pastori D Menichelli D Cammisotto V Pignatelli P. Use of direct oral anticoagulants in patients with antiphospholipid syndrome: A systematic review and comparison of the international guidelines. Front Cardiovasc Med. (2021) 8:715878. doi: 10.3389/fcvm.2021.715878 34414220 PMC8368436

[29] Pierangeli SS Harris EN. Probing antiphospholipid-mediated thrombosis: the interplay between anticardiolipin antibodies and endothelial cells. Lupus. (2003) 12:539–45. doi: 10.1191/961203303lu398oa 12892395

