# Supplementary material for: Recurrent ischemic stroke with patent foramen ovale linked to seronegative antiphospholipid syndrome: a case report and literature review
Source: Front Immunol. 2025 Apr 2;16:1558309. doi: 10.3389/fimmu.2025.1558309 (PMC12000043; doi:10.3389/fimmu.2025.1558309)

Supplementary Figure 1. The scans and images of the patient. (A) ECG showed the patient had a sinus rhythm. (B) and (C) TEE indicated normal sizing in all four chambers, without atrial septal aneurysm. (D) and (E) chest and abdominal CT showed no abnormalities. (F) and (G) the result of Doppler ultrasound of carotid artery and lower extremity Doppler ultrasounds showed no stenosis or thrombosis. ECG, electrocardiogram; TEE, transesophageal echocardiogram; PFO, patent foramen ovale.

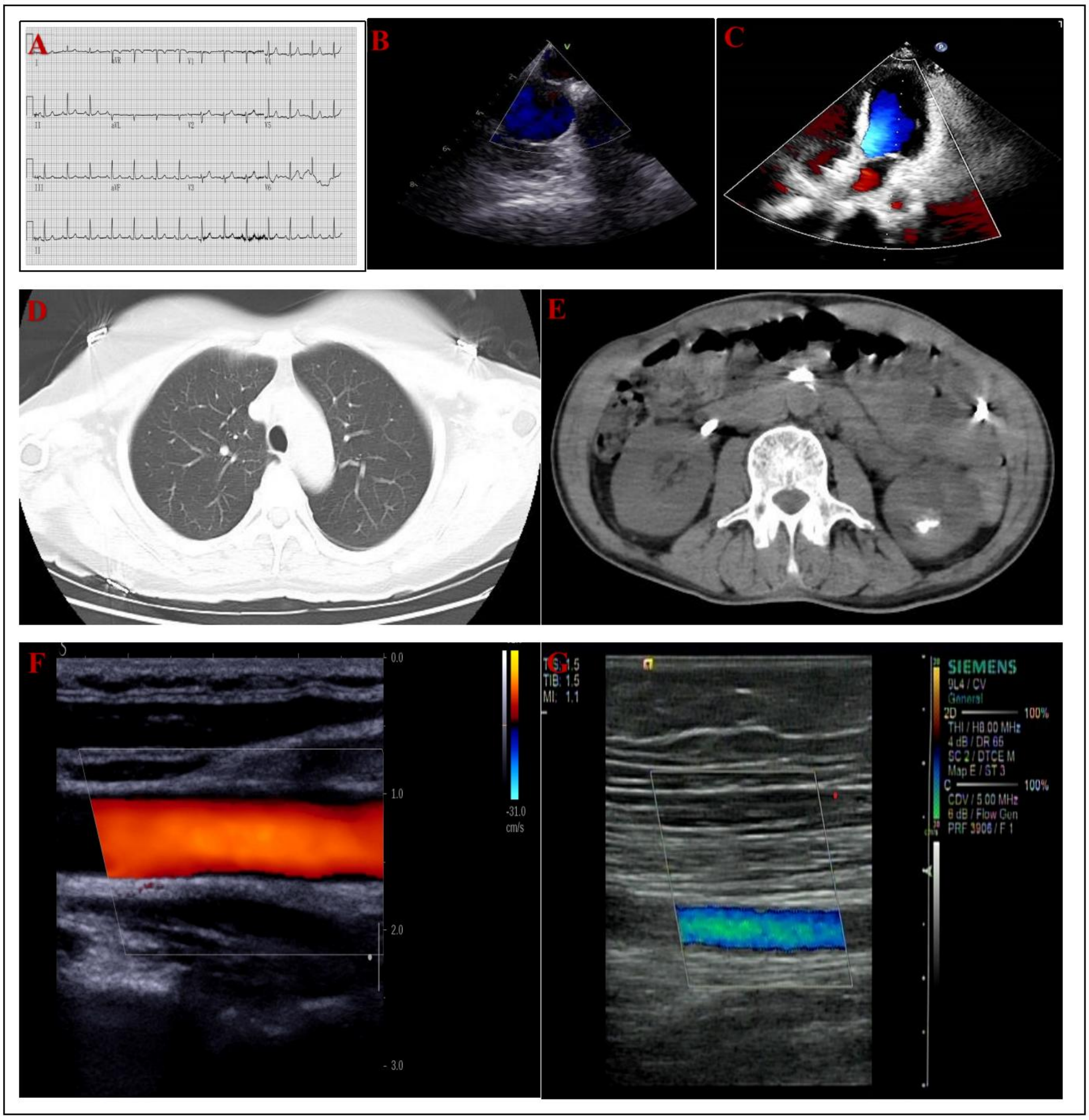

Supplement: Supplementary file 1 [file DataSheet1.pdf]
